# Bilateral Spontaneous Pneumothorax in a Patient With Metastatic Leiomyosarcoma Receiving Pazopanib: A Case Report

**DOI:** 10.7759/cureus.82161

**Published:** 2025-04-13

**Authors:** Maria Konstantinidou, Christina Chrysanthi Theocharidou, Anastasia Dimaki, Christos Emmanouilides, Fotini Ampatzidou

**Affiliations:** 1 Department of Respiratory Medicine, General Hospital of Thessaloniki "G. Papanikolaou", Thessaloniki, GRC; 2 Intensive Care Unit, General Hospital of Thessaloniki "G. Papanikolaou", Thessaloniki, GRC; 3 Department of Oncology, Interbalkan Medical Center, Pylaia, GRC

**Keywords:** adverse event, bilateral pneumothorax, leiomyosarcoma, pazopanib, pulmonary metastases, tyrosine kinase inhibitor

## Abstract

Pneumothorax can be a rare but significant adverse event in patients with sarcoma and pulmonary metastases. This case report presents an instance of bilateral pneumothorax in a patient with metastatic leiomyosarcoma treated with pazopanib. A 74-year-old man with a history of grade 3 leiomyosarcoma and lung metastases was admitted with severe respiratory distress. He had been receiving pazopanib therapy following previous treatment with high-dose ifosfamide, doxorubicin, and radiotherapy. Imaging revealed bilateral pneumothorax, and the patient subsequently experienced respiratory arrest requiring immediate resuscitation measures including needle decompression followed by chest tube placement. The patient had experienced a unilateral pneumothorax two months prior to this presentation, which had resolved with standard interventions. While the bilateral pneumothorax eventually resolved after nine days of chest tube drainage, the patient exhibited no neurological recovery following the arrest, with brain imaging revealing bilateral cortical laminar necrosis. His clinical condition deteriorated significantly after 28 days in the intensive care unit (ICU), culminating in septic shock and death. This case highlights a serious pulmonary complication that can occur during the treatment of metastatic leiomyosarcoma, particularly in patients with lung metastases. The relationship between the development of pneumothorax and pazopanib therapy, along with the challenges in management and poor clinical outcome, merits consideration when treating similar patients.

## Introduction

Pneumothorax is an uncommon but clinically significant complication with pulmonary metastases, especially in patients with sarcomas [1]. This condition is believed to stem from factors related to tumor-induced lung pathology, such as the cavitation or cystic degeneration of metastatic lesions. Though rare, cases of bilateral spontaneous pneumothorax in sarcoma patients undergoing treatment with the tyrosine kinase inhibitor (TKI) pazopanib have been documented, highlighting a potential association between the drug and pneumothorax development [2-4]. Pazopanib, approved primarily for metastatic soft tissue sarcoma, inhibits vascular endothelial growth factor (VEGF) pathways, impacting tumor growth and vascularization, but can lead to unique adverse effects such as pneumothorax, a complication scarcely reported in other TKIs of the same class [5]. Early-phase trials of pazopanib did not recognize pneumothorax as a side effect [6,7]; however, subsequent studies identified its occurrence in 1-3% of patients, primarily those with existing lung metastases [8]. With this case report, we present a patient with leiomyosarcoma and pulmonary metastases who developed bilateral pneumothorax under pazopanib therapy, exploring the mechanisms, therapeutic challenges, and implications of this rare adverse event in sarcoma management.

## Case presentation

**Figure 1:**
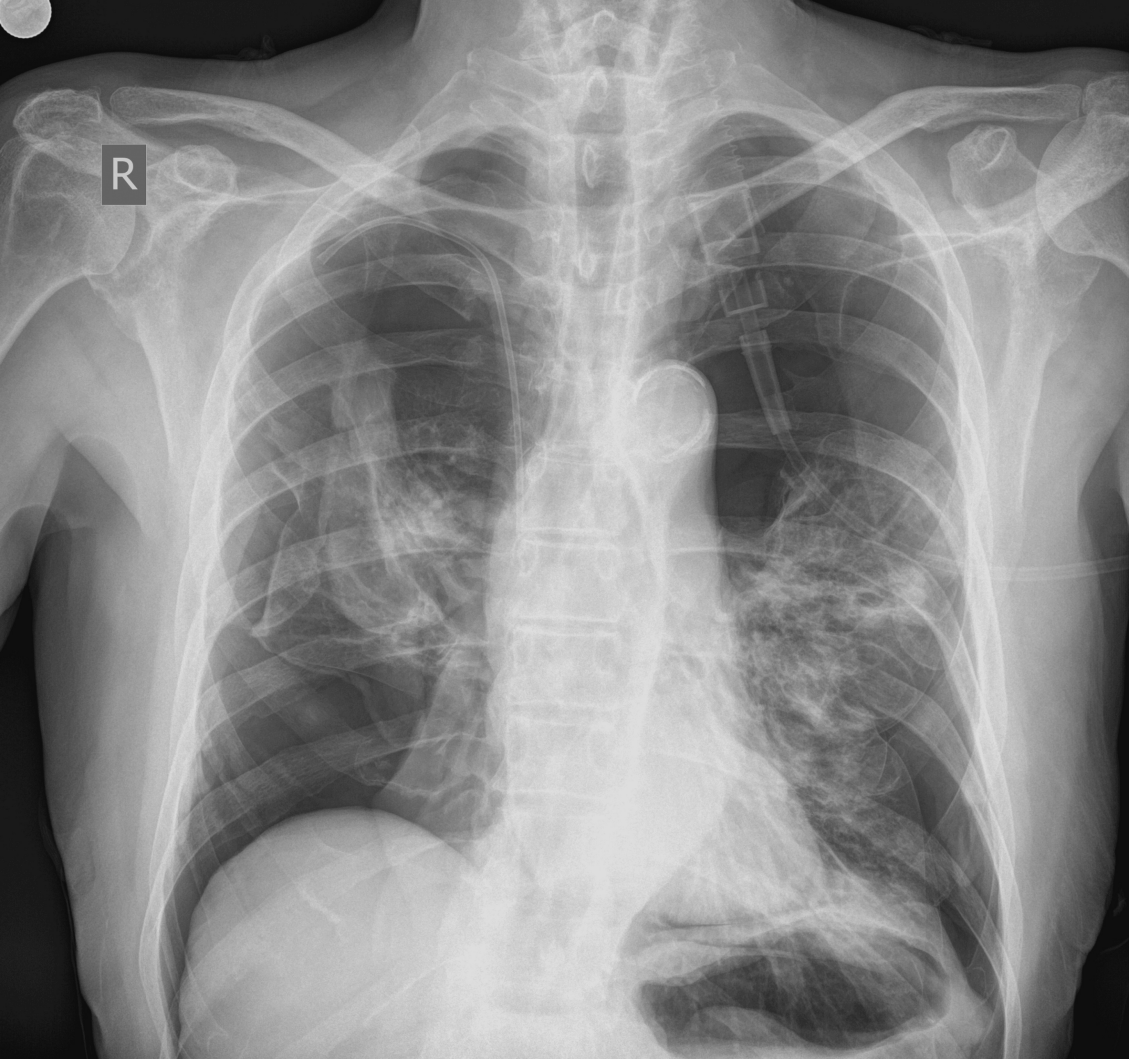
Chest X-ray of a 74-year-old male patient with leiomyosarcoma and lung metastases treated with pazopanib, demonstrating bilateral tension pneumothorax upon presentation to the emergency department.

A 74-year-old non-smoker man with a history of grade 3 leiomyosarcoma with lung metastasis was admitted to the emergency department due to dyspnea, thoracic pain, and hypoxia while initially being hemodynamically stable. The patient mentioned a fall occurring a week before his current visit. The fall involved him losing balance and experiencing a low-energy impact to the sacral region with no direct trauma to the thorax or upper extremities. Physical examination revealed respiratory distress and diminished breath sounds on lung auscultation and no evidence of chest wall contusion, tenderness, or deformity that would suggest traumatic injury. Arterial blood gas analysis indicated hypoxemia without hypercapnia or respiratory acidosis. The patient was placed on oxygen therapy and immediately underwent a chest X-ray, which revealed bilateral tension pneumothorax (Figure 1). During this evaluation, the patient quickly experienced respiratory arrest, likely due to the tension pneumothorax, prompting the immediate initiation of cardiopulmonary resuscitation (CPR). Initial pulmonary decompression was performed with bilateral needle placement at the second intercostal spaces along the midclavicular lines to relieve tension and evacuate trapped air. Following the placement of chest tubes, a significant volume of air was evacuated, allowing for successful resuscitation after 15 minutes of CPR. The patient was subsequently transferred to the hospital's intensive care unit (ICU).

**Figure 2:**
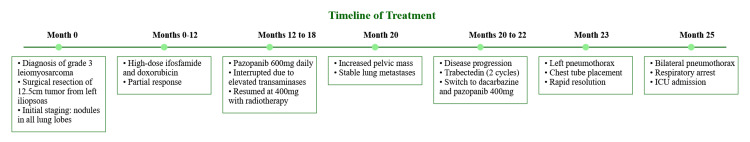
Timeline of treatment and clinical course until ICU admission. ICU: intensive care unit

In terms of medical history (Figure 2), the patient was diagnosed with grade 3 leiomyosarcoma two years prior, following the surgical resection of a 12.5 cm tumor from the left iliopsoas region. Staging at that time revealed small nodules in all lung lobes. For the following year, he underwent chemotherapy with high-dose ifosfamide and doxorubicin, achieving only a partial response, leading to the initiation of oral pazopanib at 600 mg daily. This therapy was temporarily interrupted due to elevated transaminases. After two months, imaging reassessment showed a slight increase in residual pelvic disease, but continuing improvement in pulmonary nodules. He subsequently received radiotherapy targeting the left iliopsoas and femoral vessels and resumed pazopanib at a reduced dose of 400 mg. After six months of ongoing therapy, a computed tomography (CT) scan revealed the progression of the left pelvic mass, while the lung metastases remained stable. He was treated with trabectedin; however, disease progression was evident after two cycles, leading to the initiation of third-line therapy with dacarbazine and continued pazopanib at 400 mg, achieving disease stability and demonstrating the efficacy of pazopanib without further liver toxicity. Two months prior to the presented event, the patient experienced a left-sided pneumothorax, which did not significantly impact his respiratory function and was managed with chest tube placement, resulting in rapid resolution. His treatment had not been discontinued at that time.

**Figure 3:**
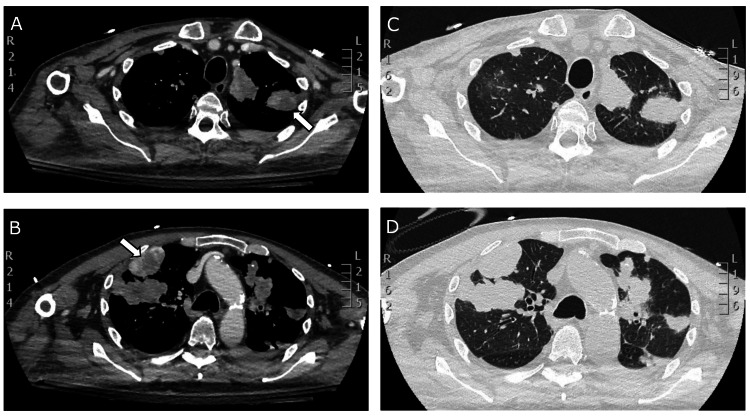
Axial chest CT images demonstrating bilateral subpleural metastatic lesions with central necrosis (arrows) on day 10 following the resolution of pneumothoraces and chest tube removal (A, B: soft tissue window; C, D: lung window). CT: computed tomography

In the ICU, initial management of the bilateral pneumothorax proved challenging, with persistent incomplete air evacuation despite chest tube placement. Although continuous air leaks were anticipated while on mechanical ventilation, complete resolution was achieved after nine days, confirmed by follow-up CT imaging (Figure 3). Upon weaning from sedation, the patient exhibited no signs of neurological recovery. While the initial head CT was unremarkable, subsequent brain MRI revealed evidence of bilateral cortical laminar necrosis, consistent with global cerebral hypoperfusion secondary to the cardiac arrest event.

The patient underwent tracheostomy on day 12 of ICU admission and was successfully weaned from mechanical ventilation to spontaneous breathing. However, despite initial cardiopulmonary stabilization, the patient's clinical condition deteriorated significantly after 28 days in the ICU, ultimately culminating in septic shock and death.

## Discussion

Pneumothorax is a recognized yet serious complication in patients with soft tissue sarcomas, especially those with pulmonary metastases [9]. Based on clinical context, pneumothorax can be traumatic, iatrogenic, and spontaneous. Spontaneous pneumothorax can be divided into primary (occurring without underlying lung disease) and secondary (associated with existing lung conditions). It can also be categorized as simple, communicating, or tension pneumothorax [10]. In our case of metastatic leiomyosarcoma, the patient developed secondary spontaneous bilateral tension pneumothorax, evidenced by the rapid progression to respiratory arrest requiring immediate decompression. Although spontaneous pneumothorax in this context is uncommon, its occurrence can significantly impact patient management and prognosis. Recent reports have demonstrated a possible association between the use of pazopanib, a TKI primarily used in metastatic renal cell carcinoma and soft tissue sarcoma, and pneumothorax, particularly in cases involving lung metastases [11]. This discussion explores the underlying mechanisms, clinical considerations, and relevant literature surrounding pneumothorax as an adverse event of pazopanib in metastatic sarcoma cases.

Apart from pazopanib therapy, several risk factors for pneumothorax can be recognized in this case. The patient reported a fall one week prior to admission, which, although it resulted in only a low-energy impact to the sacral region without direct thoracic trauma, could have possibly contributed to triggering the pneumothorax. In patients with metastatic lung disease on anti-angiogenic therapy, even minor physical stress could potentially destabilize vulnerable areas in the lung tissue. Furthermore, the patient's history of pneumothorax just two months before this presentation represents a significant risk factor. This prior event not only established the patient as having a higher susceptibility to recurrent pneumothoraces but also introduced iatrogenic factors. The previous chest tube management and subsequent healing process may have created areas of pleural adhesion or parenchymal weakening that could predispose to recurrent air leak, particularly in the setting of ongoing pazopanib therapy. These combined factors likely contributed to the severity of the bilateral presentation and subsequent respiratory arrest.

Pazopanib's mechanism of action provides insight into its unique association with pneumothorax. As a potent and selective multi-targeted TKI, pazopanib inhibits several receptors, primarily those in the VEGF family (VEGF receptor-1 (VEGFR-1), VEGFR-2, and VEGFR-3) along with platelet-derived growth factor receptors alpha and beta (PDGFR-α/β) and c-Kit [12]. This VEGF pathway inhibition leads to reduced endothelial cell migration, proliferation, and vascular permeability, ultimately compromising tumor vasculature. However, this anti-angiogenic effect can result in tissue ischemia and subsequent necrosis within tumor masses, particularly affecting pulmonary metastases [13]. Importantly, other TKIs, such as sunitinib and sorafenib, do not share this risk profile, suggesting a unique mechanism specific to pazopanib [14].

The literature describes two primary mechanisms for pazopanib-associated pneumothorax: cystic degeneration of metastatic lesions and bronchial-pleural fistula formation. In our case, imaging demonstrated subpleural metastases with necrosis (Figure 3), possibly suggesting necrosis leading to cystic degeneration as the underlying mechanism, although fistula formation could not be definitively excluded. The pathophysiological process involves two sequential steps: first, VEGF inhibition disrupts tumor vasculature, leading to ischemic necrosis and cavitation of pulmonary nodules; subsequently, the weakened necrotic tissue ruptures, creating direct communication with the pleural space. This mechanism is particularly relevant in subpleural lesions, where tissue breakdown can directly affect pleural integrity [15].

Management of pazopanib-associated pneumothorax presents significant clinical challenges, often requiring prolonged chest tube drainage and, in refractory cases, surgical interventions such as pleurectomy. The literature consistently describes difficulties in achieving complete lung re-expansion due to persistent air leakage from cavitated metastases [15]. Our case initially followed a refractory pattern, presenting with recurrent and ultimately bilateral pneumothorax. However, in contrast to previously reported cases, the air leak resolved completely after nine days of chest tube drainage, though this successful resolution of the pneumothorax did not ultimately alter the patient's poor outcome [14].

Cases of bilateral pneumothorax in patients treated with pazopanib are particularly rare [16]. A notable case series documented six patients on pazopanib who developed pneumothorax, with only one experiencing bilateral involvement. This series highlighted that patients with underlying lung metastases were at an elevated risk for pneumothorax, suggesting that the metastatic disease itself may play a more critical role in precipitating this condition than pazopanib alone [17]. Several risk factors have been identified for developing pneumothorax in this patient population, including larger lung metastases (≥30 mm), history of pneumothorax (as seen in our patient), multiple tumors, cavitary or pleural-based nodules, and longer duration of pazopanib treatment [11,18]. Additionally, even minor trauma may serve as a triggering event in patients with already compromised lung parenchyma, as possibly seen in our case, though the patient's fall did not involve direct thoracic impact. The reported incidence of pazopanib-associated pneumothorax is approximately 3% among sarcoma patients [13], although early trials did not recognize this complication [6,7]. The evolution in understanding this adverse effect emphasizes the importance of post-marketing surveillance in identifying rare but significant complications.

Several limitations should be acknowledged in the context of this case report. First, there is a paucity of studies investigating the dose-dependent relationship between pazopanib and the risk of pneumothorax development. Whether higher doses consistently correlate with increased risk remains unknown. Second, the relationship between the duration of pazopanib administration and pneumothorax risk has not been thoroughly characterized in the literature, making it difficult to establish optimal treatment timelines for high-risk patients. Third, as a single case report, our findings cannot establish definitive causal relationships or risk assessments, though they add to the growing body of evidence regarding this rare but serious complication.

Prevention strategies that may warrant consideration include more intensive radiological surveillance for patients with known risk factors, particularly those with subpleural metastases, prior pneumothorax, or cavitary lesions. In some cases, temporary interruption or dose reduction of pazopanib might be considered following the initial pneumothorax, though evidence supporting this approach is limited. Development of institutional protocols for the early identification and management of pneumothorax in this patient population could potentially improve outcomes and reduce morbidity.

## Conclusions

This case illustrates several important aspects of pazopanib-associated pneumothorax in sarcoma patients. While pazopanib remains a valuable therapeutic option for metastatic soft tissue sarcoma, its unique mechanism of action through VEGF pathway inhibition can lead to serious pulmonary complications. The development of bilateral pneumothorax in our patient highlights both the potential severity of this adverse event and the complex management challenges it presents. The case particularly emphasizes the importance of recognizing risk factors, such as subpleural metastases with cavitation, which may predispose patients to this complication. Clinicians should also keep in mind that trivial trauma might occasionally serve as a contributing factor, despite the fact that the trauma pattern was not consistent with direct chest impact in our patient. Given that pazopanib is the only TKI in its class associated with this adverse effect, clinicians should maintain heightened vigilance when treating patients with pulmonary metastases. Regular radiological monitoring, early recognition of warning signs, and prompt intervention may help optimize outcomes. Further research is needed to identify predictive factors for pneumothorax development and to establish evidence-based management protocols for this patient population.
